# Association between alcohol consumption and mild cognitive impairment: A protocol of dose–response meta-analysis

**DOI:** 10.1097/MD.0000000000016098

**Published:** 2019-07-05

**Authors:** Xu Hui, Jing Li, Yongfeng Lao, Bibo Jia, Lijuan Hou, Zhenxing Lu, Qinghong Gu, Junqiang Niu, Hairong Bao, Peijing Yan, Liang Yao

**Affiliations:** a School of Public Health, Lanzhou University; b Second Clinical Medical College of Lanzhou University; c First Clinical Medical College of Lanzhou University; d Evidence-Based Medicine Center of Lanzhou University; e The First Hospital of Lanzhou University; f Institute of Clinical Research and Evidence Based Medicine, Gansu Provincial Hospital, Lanzhou; g Chinese Medicine Faculty of Hong Kong Baptist University, Kowloon Tong, Hong Kong, China.

**Keywords:** alcohol, dose–response, meta-analysis, mild cognitive impairment, protocol

## Abstract

**Objective:**

The objective of this study is to investigate the potential dose–response association between alcohol consumption and the risk of mild cognitive impairment (MCI).

**Methods:**

We will perform a dose–response meta-analysis (DRMA) of cohort studies to explore the dose–response relationship between alcohol intake and MCI. A comprehensive literature search of PubMed, EMBASE, The Cochrane Library, Chinese BioMedical Literature Database (CBM), China National Knowledge Infrastructure (CNKI), VIP, and Wan-Fang Database will be conducted. Two investigators will independently select studies, extract data, and assess the quality of the included study. The Newcastle-Ottawa Scale will be used to assess the quality of include studies. The Grading of Recommendations Assessment, Development, and Evaluation (GRADE) system and A MeaSurement Tool to Assess systematic Reviews (AMSTAR) will be used to assess the quality of evidence and methodological quality. Any disagreement will be resolved by the third investigator. We will use the hazard ratio as the effect indicator, and piecewise linear regression model and restricted cubic spline model will be used for linear and nonlinear trend estimation, respectively. There is no requirement of ethical approval and informed consent.

**Discussion:**

This is the first DRMA to explore the dose–response relationship between alcohol intake and MCI. We predict it will provide high-quality evidence to prevent clinical MCI and dementia.

**Registration:**

The DRMA is registered in the PROSPERO (CRD42019127261) international prospective register of systematic review.

## Abstract 2

Supplemental Digital Content is available in the text

## 1 Introduction

Mild cognitive impairment (MCI) was defined as the intermediate stage between normal age-related impairment in cognitive abilities and the progression of Alzheimer's disease (AD) with a deterioration of memory, attention, and cognitive function.^[1,2]^ An updated American Academy of Neurology (ANN) guideline reported that the prevalence of MCI from 60 to 84 years old was between 6.7% and 25.2%, and with a continuous rising trend.^[3]^ Moreover, the cumulative incidence of dementia was 14.9% in senile patients with MCI.^[3]^ As no curable intervention was found for dementia, prevention for MCI has increasingly caught the attention of the field in the past decade.^[4,5]^

The relationship between alcohol intake and MCI in the original study is still controversial.^[6–8]^ A cohort study revealed participants who did not drink alcohol and those who drank alcohol frequently at midlife were twice as likely to have MCI more than those who drank alcohol infrequently. However, no significant association was found between alcohol intake and the incidence of MCI in another 2 cohort studies.

In addition, high-quality meta-analysis has been increasingly regarded as one of the key tools for achieving evidence,^[9,10]^ and dose–response meta-analysis (DRMA) has higher quality.^[11]^ To the best of our knowledge, there is no DRMA to investigate the association between alcohol intake and MCI. Therefore, we conduct this DRMA to better quantify the association between alcohol consumption and risk of MCI to prevent clinical MCI and dementia.

## 2 Method

This DRMA had applied for registration in the PROSPERO (CRD42019127261) international prospective register of systematic review. The study will be conducted according to the Preferred Reporting Items for Systematic reviews and Meta-Analysis Protocols (PRISMA-P) guidelines and G-dose checklist.^[12–15]^ The Grading of Recommendations Assessment, Development, and Evaluation (GRADE) system will be used to quantify absolute effects and quality of evidence.^[16]^ A MeaSurement Tool to assess systematic Reviews (AMSTAR) will be used to assess methodological quality.^[17,18]^ No requirement of ethical approval and informed consent is needed because it is a retrospective study.

### 2.1 Search strategy

We will conduct a systematic search without language and year restrictions to identify all relevant published studies. The following electronic databases will be searched: PubMed, EMBASE, The Cochrane Library, Chinese BioMedical Literature Database (CBM), China National Knowledge Infrastructure (CNKI), VIP, and Wan-Fang Database. The search strategy will include key words relating to the intervention (alcohol) and the disease (MCI). References of included study will also be traced back to find potential qualified studies. To identify other relevant study data, we will contact the authors of published studies for incomplete data. The detailed procedure of literature search could be found in Appendix.

### 2.2 Inclusion criteria

We will include studies that met the following criteria:

(1) investigated the association between MCI (recommendation by the International Working Group on Mild Cognitive Impairment) and alcohol intake^[19]^;
(2) alcohol intake was categorized into ≥3 levels and drinking levels can be quantified;
(3) acquired directly or indirectly about level-specific hazard ratio (HR)/relative risk (RR) and 95% confidence interval (CI);
(4) cohort studies.

### 2.3 Exclusion criteria

Studies will be excluded if one of the following conditions is met:

(1) the research data were not complete;
(2) the study were reviews, comments, letters, conference abstracts, editorials, systematic reviews or meta analyses, protocols, case reports, cross-section studies, nonhuman studies, case–control studies, case series, and briefs;
(3) the study was not in Chinese or English;
(4) if multiple articles were published based on the same cohort, we chose the study with longer follow-up or a larger sample size.

### 2.4 Study selection

Two investigators will independently select the studies and any disagreement will be resolved by a third investigator through discussion. Titles and abstracts of the studies retrieved by the literature search will be screened based on inclusion/exclusion criteria, and we will acquire the full text of potentially relevant studies for further assessment.

### 2.5 Data extraction

A standard form will be used to extract data from the included studies. Two investigators will independently extract the related data and any dispute will be discussed and resolved by a third investigator. Missing data will be requested from study authors. Extracted information will include

(1) basic information: author, publication year, country and setting, source of funding;
(2) participants: diagnostic criteria, the sample size of male in each group, age, sex, length of follow-up, withdrawals, or losses to follow-up;
(3) interventions: details of alcohol consumption;
(4) outcomes: the occurrence of MCI, HR/RR, and 95% CI.

### 2.6 Quality evaluation

The Newcastle-Ottawa Scale (NOS) will be used to evaluate the quality of the cohort studies included in this DRMA.^[20]^ The NOS is based on selection (4 items), comparability (1 item), and outcome (3 items). We will consider 0 to 3, 4 to 6, and 7 to 9 stars as low, moderate, and high quality of study, respectively. Two review authors will independently assess the quality of the included studies. If there is any question, a third investigator will solve it through discussion.

### 2.7 Statistical analysis and assessment of heterogeneity

The statistical analyses will be conducted to use STATA version 12.0 (STATA Corp, College Station, TX), with two-tailed *P* < .05 for statistical significance.

Piecewise linear regression model and restricted cubic spline (RCS) model will be used for nonlinear trend estimation; a flexible meta-regression based on RCS function will be used to fit the potential nonlinear trend; and generalized least-square method will be used to estimate the parameters.^[21]^ The HR and 95% CI is used as the pooled effect size for time-to-event data, and RR and 95% CI is used as the effect indicator for other dichotomy variables.

*Q* test and *I*^2^ statistic will be used to evaluate heterogeneity of studies. The level of significance was set equal to 0.05 for the *Q* test. *I*^2^ values ≤25%, 25% to 50%, 50% to 75%, and >75% indicated no, small, moderate, and significant heterogeneity, respectively. When significant heterogeneity exists, a random-effects model will be used. Otherwise the fixed-effect model will be used. If there is a high heterogeneity between studies, subgroup analysis and sensitivity analysis will be used to explore the heterogeneity.

### 2.8 Subgroup analysis and sensitivity analysis

We will perform a subgroup analysis using the following group variables:

(1) sex;
(2) types of study (perspective study and retrospective study);
(3) types of alcohol.

## 3 Discussion

There are some problems and limitations in this DRMA. First, the original studies have different units of alcohol intake such as infrequently (less than once a month),^[6]^ drinks per day/week,^[22,23]^ light-to-moderate consumption,^[24]^ or former/current alcohol consumption,^[25,26]^ which cause difficulties to integrate information from original studies. Then, this DRMA will be limited by insufficiently reporting some important details such as type of alcohol, amount, and duration of consumption.^[27]^ Another possible limitation is that the original studies mainly rely on the self-report questionnaire to assess alcohol intake; they may cause measurement bias especially for MCI patients.

For the measurement of alcohol intake, we plan to perform the DRMA base on the alcohol intake measured by amount (drinks/wk and g/d) and frequency (times/wk). The final report of the meta-analysis in the form of scientific paper will be published in peer-reviewed journals.

## Author contributions

**Conceptualization:** Hairong Bao, Peijing Yan, Liang Yao.

**Data curation:** Xu Hui, Jing Li, Bibo Jia, Lijuan Hou, Junqiang Niu, Liang Yao.

**Formal analysis:** Xu Hui, Jing Li, Yongfeng Lao, Hairong Bao.

**Investigation:** Yongfeng Lao, Lijuan Hou, Liang Yao.

**Methodology:** Xu Hui, Jing Li, Qinghong Gu, Peijing Yan.

**Project administration:** Hairong Bao, Peijing Yan, Liang Yao.

**Software:** Xu Hui, Jing Li, Bibo Jia, Zhenxing Lu, Peijing Yan.

**Supervision:** Hairong Bao, Peijing Yan, Liang Yao.

**Writing – original draft:** Xu Hui, Jing Li, Yongfeng Lao.

**Writing – review and editing:** Bibo Jia, Lijuan Hou, Zhenxing Lu, Qinghong Gu, Junqiang Niu, Hairong Bao, Peijing Yan, Liang Yao.

## Supplementary Material

Supplemental Digital Content
