# Supplementary material for: Association between alcohol consumption and mild cognitive impairment: A protocol of dose–response meta-analysis
Source: Medicine (Baltimore). 2019 Jul 5;98(27):e16098. doi: 10.1097/MD.0000000000016098 (PMC6635151; doi:10.1097/MD.0000000000016098)
Supplement: Supplemental Digital Content [file medi-98-e16098-s001.doc]

Appendix

1. PubMed

((((((((((((((((((ethanol[Title/Abstract]) OR alcohol*[Title/Abstract]) OR blood alcohol level[Title/Abstract]) OR blood alcohol content[Title/Abstract]) OR wine*[Title/Abstract]) OR liquor*[Title/Abstract]) OR spirit*[Title/Abstract]) OR beer*[Title/Abstract]) OR beverage*[Title/Abstract]) OR alcohol drinking[Title/Abstract]) OR drinking behavior[Title/Abstract]) OR alcohol consumption[Title/Abstract]) OR alcohol intake[Title/Abstract]) OR drink*[Title/Abstract]) OR drunk*[Title/Abstract])) OR (((("Ethanol"[Mesh]) OR "Blood Alcohol Content"[Mesh]) OR "Alcohol Drinking"[Mesh]) OR "Drinking Behavior"[Mesh]))) AND ((((((((((((((((((((((((((((((((((((((mild cogniti* impairment*[Title/Abstract]) OR mild cogniti* defect*[Title/Abstract]) OR mild cogniti* dysfunction*[Title/Abstract]) OR mild neurocogniti* disorder*[Title/Abstract]) OR mild cogniti* defici*[Title/Abstract]) OR mild cogniti* declin*[Title/Abstract]) OR prodrom* dement*[Title/Abstract]) OR MCI[Title/Abstract]) OR N‐MCI[Title/Abstract]) OR A‐MCI[Title/Abstract]) OR M‐MCI[Title/Abstract]) OR nMCI[Title/Abstract]) OR aMCI[Title/Abstract]) OR mMCI[Title/Abstract]) OR pre-clinical dement*[Title/Abstract]) OR preclinical dement*[Title/Abstract]) OR preclinical Alzheimer*[Title/Abstract]) OR pre‐clinical Alzheimer*[Title/Abstract]) OR preclinical AD[Title/Abstract]) OR pre‐clinical AD[Title/Abstract]) OR age-associated memory impairment*[Title/Abstract]) OR AACI[Title/Abstract]) OR CIND[Title/Abstract]) OR ACMI[Title/Abstract]) OR ARCD[Title/Abstract]) OR SMC[Title/Abstract]) OR CIND[Title/Abstract]) OR BSF[Title/Abstract]) OR AAMI[Title/Abstract]) OR LCD[Title/Abstract]) OR QD[Title/Abstract]) OR AACD[Title/Abstract]) OR MNCD[Title/Abstract]) OR MCD[Title/Abstract]) OR clinical dementia rating scale 0.5[Title/Abstract]) OR CDR 0.5[Title/Abstract])) OR "Cognitive Dysfunction"[Mesh])

1. EMBASE

#7 #3 AND #6

#6 #4 OR #5

#5 'mild cognitive impairment':ab,ti OR 'mild cognitive defect':ab,ti OR 'mci':ab,ti OR 'n‐mci':ab,ti OR 'a‐mci':ab,ti OR 'm‐mci':ab,ti OR 'nmci':ab,ti OR 'amci':ab,ti OR 'mmci':ab,ti OR 'pre-clinical dementia':ab,ti OR 'preclinical dementia':ab,ti OR 'mild neurocognitive disorder':ab,ti OR 'preclinical alzheimer':ab,ti OR 'pre‐clinical alzheimer':ab,ti OR 'preclinical ad':ab,ti OR 'pre‐clinical ad':ab,ti OR 'age-associated memory impairment':ab,ti OR 'aaci':ab,ti OR 'acmi':ab,ti OR 'arcd':ab,ti OR 'smc':ab,ti OR 'cind':ab,ti OR 'bsf':ab,ti OR 'aami':ab,ti OR 'lcd':ab,ti OR 'qd':ab,ti OR 'aacd':ab,ti OR 'mncd':ab,ti OR 'mcd':ab,ti OR 'mild cognitive deficit':ab,ti OR 'mild cognitive decline':ab,ti OR 'prodromal dementia':ab,ti OR 'clinical dementia rating scale 0.5':ab,ti OR 'cdr 0.5':ab,ti

#4 'mild cognitive impairment'/exp OR 'mild cognitive defect'/exp

#3 #1 OR #2

#2 'ethanol':ab,ti OR 'alcohol*':ab,ti OR 'blood alcohol level':ab,ti OR 'blood alcohol content':ab,ti OR 'wine*':ab,ti OR 'liquor*':ab,ti OR 'spirit*':ab,ti OR 'beer*':ab,ti OR 'beverage*':ab,ti OR 'alcohol drinking':ab,ti OR 'drinking behavior':ab,ti OR 'alcohol consumption':ab,ti OR 'alcohol intake':ab,ti OR 'drink*':ab,ti OR 'drunk*':ab,ti

#1 'alcohol'/exp OR 'alcohol blood level'/exp OR 'drinking behavior'/exp OR 'alcohol consumption'/exp

1. CBM

#1 "饮酒"[不加权:扩展]

#2 ((("饮酒"[常用字段:智能]) OR "喝酒"[常用字段:智能]) OR "酒精"[常用字段:智能]) OR "酒"[常用字段:智能]

#3 (#3) OR (#2)

#4 (("轻度认知障碍"[常用字段:智能]) OR "轻度认知损伤"[常用字段:智能]) OR "MCI"[常用字段:智能]

#5 (#5) AND (#4)
